# Localized delivery of non-viral gene-bearing nanoparticles into the rat brain following focused ultrasound-mediated BBB opening

**DOI:** 10.1186/2050-5736-3-S1-P29

**Published:** 2015-06-30

**Authors:** Brian Mead, Panagiotis Mastorakos, Jung Soo Suk, Ji Song, Justin Hanes, Richard Price

**Affiliations:** 1 University of Virginia, Charlottesville, Virginia, United States; 2 Johns Hopkins University, Baltimore, Maryland, United States

## Background/introduction

By preventing more than 98% of currently used pharamaceutical agents from entering the brain, the blood-brain barrier critically reduces the ability of therapeutics to treat a variety of central nervous system (CNS) disorders including glioblastoma and neurodegenerative diseases. Focused ultrasound (FUS) mediated microbubble oscillation and subsequent blood brain barrier (BBB) permeabilization has been explored as a powerful non-invasive strategy for the delivery of circulating therapeutic agents into the CNS. FUS in conjunction with microbubbles has been shown to facilitate non-damaging, reversible and localized disruption of the BBB, leading to substantial increases in nanoparticle (NP) concentrations in ultrasound-treated tissue. Once beyond the BBB, the extracellular matrix acts as a steric and adhesive barrier and limits NP distribution. Coating sub-100 nm nanoparticles with a dense brush layer of polyethylene glycol (PEG) to limit the interactions with the ECM leads to a significant improvement of their diffusivity in brain tissue. The current study investigates the ability of FUS to deliver densely PEGylated brain penetrating cationic polymer-based gene vectors across the BBB to mediate robust and long-term transgene expression.

## Methods

Anesthetized 200g Sprague-Dawley rats were secured in a stereotaxic frame and their heads were depilated. A 1.5-inch single element 1MHz focused ultrasound transducer was ultrasonically coupled. The tail vein was cannulated, and a coinjection of microbubbles (105/g) and 50 nm PEGylated polyethylenimine (PEG-PEI) nanoparticles (50 ug, 100 ug, 200 ug or 350 ug doses) immediately preceded ultrasound treatment. We delivered luciferase and mCherry transgenes under control of a beta-actin promoter. All sonications were performed with a 0.5% duty cycle, a total time of two minutes, and a peak negative pressure of 0.6 MPa. Luciferase expression was assessed through bioluminescent imaging following a 150 mg/kg injection of luciferin in an *In Vivo* Imaging System. Following euthanasia, animals were perfused with saline, and brains were dehydrated and cryosectioned. Mounted sections were stained with mCherry, Draq5 or H&E.

## Results and conclusions

**Figure 1:**
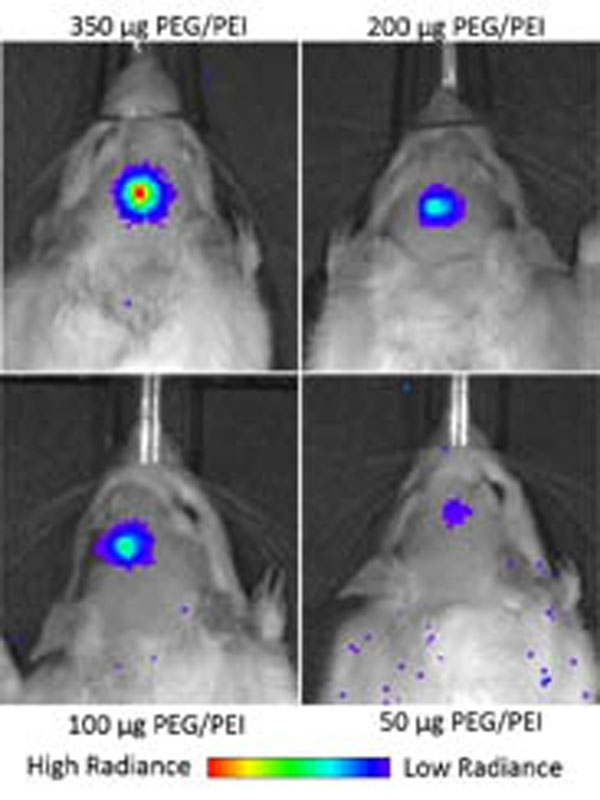
FUS mediated delivery of luciferase-bearing PEG-PEI NP mediates targeted, dose-dependent bioluminescence in a rat.

Delivery of luciferase-bearing PEG-PEI NP resulted in robust bioluminescence through day 28, the last day tested. Bioluminescence was dose-dependent (Figure 1). Under H&E, no signs of histological defect were found at the lowest two doses administered, while the 200 ug dose had only a single site of minor damage. In order to assess transfection efficiency rat brains treated with FUS and 200 ug of mCherry-expressing PEG-PEI seven days prior were sectioned and stained with a marker for cell nuclei. 41% of cells in the FUS-treated region were found to express the mCherry reporter gene compared to only 6% on the contralateral non-treated region. FUS mediated BBB disruption presents a novel platform for drug and gene delivery technologies to the brain. Our results indicate that robust gene expression can be achieved by systemically delivering densely PEGylated cationic polymer gene vectors and that transgene expression can be maintained for at least 28 days. Currently, we are analyzing the therapeutic efficacy of FUS-mediated gene therapy in a rat model of neurodegenerative disease.

